# Emotion topology: extracting fundamental components of emotions from text using word embeddings

**DOI:** 10.3389/fpsyg.2024.1401084

**Published:** 2024-10-08

**Authors:** Hubert Plisiecki, Adam Sobieszek

**Affiliations:** ^1^Research Lab for the Digital Social Sciences, IFIS PAN, Warsaw, Poland; ^2^Department of Psychology, University of Warsaw, Warsaw, Poland

**Keywords:** word embeddings, emotion decomposition, natural language processing, valence, arousal

## Abstract

This exploratory study examined the potential of word embeddings, an automated numerical representation of written text, as a novel method for emotion decomposition analysis. Drawing from a substantial dataset scraped from a Social Media site, we constructed emotion vectors to extract the dimensions of emotions, as annotated by the readers of the texts, directly from human language. Our findings demonstrated that word embeddings yield emotional components akin to those found in previous literature, offering an alternative perspective not bounded by theoretical presuppositions, as well as showing that the dimensional structure of emotions is reflected in the semantic structure of their text-based expressions. Our study highlights word embeddings as a promising tool for uncovering the nuances of human emotions and comments on the potential of this approach for other psychological domains, providing a basis for future studies. The exploratory nature of this research paves the way for further development and refinement of this method, promising to enrich our understanding of emotional constructs and psychological phenomena in a more ecologically valid and data-driven manner.

## 1 Introduction

In the study of core components of emotions various methods have been used. A large number of studies focus on the core components of emotions by using controlled environments. Here, participants either annotate distinct stimuli, such as photos of facial expressions (Calder et al., 2001; Fontaine et al., 2002, 2007; Schlosberg, 1952; Shaver et al., 1987) or assess their emotional experiences through structured questionnaires (Nowlis and Nowlis, 1956; Feldman, 1995; Stanisławski et al., 2021). These studies have explored areas such as facial expressions, emotion terms, and self-reported emotional experiences. Except for self-reports, participants annotate stimuli based on their emotional resonance. For instance, a photo capturing a broad Duchenne smile might receive a maximum rating for inferred happiness (Calder et al., 2001; Ekman et al., 1990; Tseng et al., 2014). Other research, following the Multidimensional Scaling (MDS) approach, requires participants to gauge the emotional similarity among various stimuli, such as musical pieces (Dellacherie et al., 2011), emotion terms (Bliss-Moreau et al., 2020), and facial expressions (Woodard et al., 2022).

To analyze these core components, researchers frequently utilize Principal Component Analysis (PCA) (e.g., Calder et al., 2001; Feldman, 1995; Fontaine et al., 2007; Lampier et al., 2022). At its core, PCA condenses intricate datasets by converting correlated variables into a smaller set of uncorrelated ones, known as principal components. These components highlight the primary patterns within the data (Abdi and Williams, 2010). When applied to emotional experience studies, PCA effectively pinpoints foundational dimensions like valence. It does so by transforming extensive emotional descriptors (e.g., scores from an emotional experience questionnaire) into distinct, principal emotional axes (e.g., positive–negative). This method provides researchers with a refined lens to understand the complex landscape of human emotions.

Through statistical analysis, psychologists have proposed various models of the core structure of emotional experience. These models often suggest two primary dimensions: valence (e.g., happiness vs. sadness) and arousal (e.g., stressed vs. relaxed) (Russell, 1980; Stanisławski et al., 2021). Some models also introduce additional dimensions like potency/dominance, which gauges how in control individuals feel over their environment and others (e.g., anger—high dominance; fear—low dominance), and unpredictability, reflecting the consistency of one’s surroundings in eliciting emotions (e.g., surprise—high unpredictability; calmness—low unpredictability) (Fontaine et al., 2007; Mehrabian, 1996; Russell and Mehrabian, 1977). Nonetheless, certain researchers continue to advocate for a strictly 2-dimensional perspective (Bliss-Moreau et al., 2020).

The dimensional framework, despite some disagreements about its structure, has gained substantial support in the psychological community. It’s been incorporated into neuroscientific research, offering fresh perspectives on emotional processing in the brain (Posner et al., 2005) and the origins of depression (Barrett et al., 2016). This approach has proven effective in gauging affect in physical activities (for a comprehensive review, refer to Evmenenko and Teixeira, 2022), advertising (Wiles and Cornwell, 1991), various priming and linguistic investigations (Imbir, 2016; Imbir et al., 2020; Syssau et al., 2021; Yao et al., 2016), and in machine learning (Islam et al., 2019; Martínez-Tejada et al., 2020; Nicolaou et al., 2011). While an exhaustive discussion of the dimensional model’s applications is beyond this article’s scope, we want to emphasize its broad appeal, not only within psychology but also in other scientific disciplines.

Our paper introduces a data-driven method that utilizes word embeddings (a machine learning technique) to analyze emotional expression as communicated and perceived through the medium of text and extract its core dimensions from vast amounts of text that reflect real-world contexts. Innovations in word embeddings facilitate the quantitative examination of extensive text datasets (Mikolov et al., 2013a,b). By automating insight extraction from texts, these embeddings have the potential to replicate previous findings in a new medium—unprompted written text—garnering more objective evidence for their validity. Furthermore, they can process vast text volumes, expanding the impact of conclusions drawn (Jackson et al., 2022). In subsequent sections, we offer a comprehensive review of word embeddings and discuss their potential benefits. We then transition into the details of our current study. Prior to presenting the methodology, we also establish clear definitions for the concepts associated with word embeddings, ensuring they are well anchored in emotion research.

Word embeddings are a technique popularized by Mikolov et al. (2013a,b) which makes it possible to quantify natural language. It computes separate strings of numbers (usually between 100 and 500 long), known as vectors, for each unit of text that is to be analyzed. Most often the units are words (hence “word” embeddings), and so each unique word in a given text gets assigned a vector which encodes its relation to the other words and can therefore be used to analyze its properties (Gutiérrez and Keith, 2019). In the case where one wants to analyze whole documents, composed of multiple words, separate vectors can be created for each of them as well (Le and Mikolov, 2014).

Some of the popular traits of these vectors are that, given that they were derived from a large enough batch of text (the more the better), their similarity (calculated through a formula called cosine similarity) correlates with human judgements about the similarity of the words that they relate to (Jatnika et al., 2019). Their results are therefore similar to the results obtained through the MDS method, providing a similarity metric that replaces human judgments made in the laboratory.

Importantly, these word embeddings have been used repeatedly to predict (using simple techniques, such as linear regressions) different meanings of text snippets. These use cases included, among others, predicting diseases based on the International Classification of Diseases (ICD-10) and the Unified Medical language System (UMLS) (Khattak et al., 2019), identifying cultural biases (Charlesworth et al., 2021; Durrheim et al., 2023), human judgements (Richie et al., 2019), moral values (Lin et al., 2018), and emotions and sentiments (Al-Amin et al., 2017; Jia, 2021; Plisiecki and Sobieszek, 2023; Widmann and Wich, 2022). This last application of word embeddings is especially important for the current study as it shows that word embeddings encode information that correlates with emotional meanings. This case is further strengthened by van Loon and Freese’s (2023) research, which has directly shown that affective meaning can be recovered from word embeddings by successfully predicting evaluation, potency, and activity profiles of words. Al-Amin and his team (2017) predicted positive vs. negative sentiment of texts collected from Bengalese blogging websites. Jia (2021) classified both basic emotions and overall polarity in Chinese texts. Plisiecki and Sobieszek (2023) showed that leveraging advanced word embeddings makes it possible to predict a range of emotional indices for singular words in different languages (English, German, French, Polish, Dutch). Widmann and Wich (2022) prepared a comparison of different ways of creating word embeddings on German texts for the prediction of basic emotions, comparing both newer and more classical approaches of constructing them and showed that all of them have significant predictive ability. These examples stand as evidence that word embeddings encode emotional information. They are therefore good sources of data for the current application.

Think of creating word embeddings as mapping words to a multidimensional space where the location of each word is determined by its context, or the words with which it often coexists. Imagine a large book, where every unique word is listed. The creation of word embeddings begins with each word starting at a random location in this space. As we move through the book, sentence by sentence, the algorithm adjusts the positions of the words in this space based on their context. For instance, if “cat” and “kitten” often appear in similar contexts, they gradually move closer together. Conversely, “cat” and “refrigerator”, unlikely to share much context, would drift apart. This process is repeated multiple times (known as iterations) on the entire book, refining the word positions each time. After sufficient iterations, the distances and angles between word vectors represent different types of semantic and syntactic similarities. For instance, words with similar meanings would be closer together, and the direction of specific relations (such as verb tense or gender) would be consistent. This way, word embeddings provide a rich, numeric interpretation of word relationships, useful in various language-related tasks (Mikolov et al., 2013a,b).

These word-level embeddings can be extended to document-level representations. Le and Mikolov (2014) introduced the Paragraph Vector, or Doc2Vec, an extension of word2vec that computes a vector for a sentence or document, not only for individual words. The technique involves training a model where the document vector, along with the word vectors, work together to predict the surrounding words in a document, thereby capturing the semantic essence of the entire text. Just like single words move closer or further in this numerical space based on their cooccurrences with other words, so too now whole documents get embedded in places where they fit best based on the similarities and differences in their overall content and context. This document-level vector enables researchers to compare and contrast entire documents, opening up further avenues in natural language processing tasks.

In this study we explore whether similar emotional components to those identified in previous literature (e.g., Fontaine et al., 2002), can be extracted from a large text dataset using word embeddings. We reverse the process of annotation and make use of a dataset in which the participants did not describe emotions using questionnaires, but rather spotted them in an already existing array of natural language expressions. While describing human emotions using questionnaires is not an everyday task for human beings, and therefore is not natural to them, potentially leading to issues of ecological validity, the action of inferring emotions from language is an everyday, nearly constant exercise that humans engage in. Furthermore, this specific type of judging others’ emotions—through text written by a stranger—is a very common occurrence in today’s digital world, and therefore is of high importance to the research community. Using word embeddings, we represent the annotated texts in an emergent numerical space.

In the following text, we will use a specific terminology for describing different concepts related to word embeddings, as applied to the study of emotion. This is done to enhance clarity and provide psychologists with a strong conceptual grasp of the following study. 1. To describe the multidimensional space, within which numerical vectors reside, we will use the term Emotional Space. 2. The vectors representing the emotional content of texts will be called *Emotion Vectors*. 3. When vectors do not correspond to specific emotions, but to words or single documents we will use either *Word Vectors* or *Document Vectors*, to designate them.

## 2 Method

### 2.1 Dataset

The GoEmotions dataset was developed by a team of researchers at Google to study human emotions within the realm of machine learning (Demszky et al., 2020). It includes 58,000 Reddit comments annotated with regard to 28 unique emotions, totaling over 210,000 annotations. The data came from a Reddit data dump, sourced from the reddit-data-tools project. The data dump included all comments from 2005 to January 2019. As the Reddit platform is composed of different communities of users, called Subreddits, all communities with at least 10 k comments were chosen for the analysis. The comments from different subreddits were then further balanced. First, the number of comments from the most popular subreddits was capped at the median Subreddit count. The comments were then randomly sampled for annotation.

Because the Reddit community does not reflect the globally diverse population, due to a skew towards offensive language, the toxic comments were removed from the dataset using a pre-defined list of offensive words and the help of manual annotators. This was done before the sampling process. According to best practices the researchers have modified the dataset by removing stop words and stemming the words in order to transform them into their base form (e.g., “fearsome” into “fear”).

### 2.2 Emotion taxonomy

The emotion taxonomy for annotation was created as a result of three steps: 1. Manual annotation of a small subset of the data to ensure proper coverage of emotions expressed in the text. 2. Review of psychological literature on basic emotions (Plutchik, 1980; Cowen and Keltner, 2020; Cowen et al., 2019). 3. Removal of the emotions that were deemed to have a high overlap to limit the overall number of emotions.

The resulting list of emotions included: *admiration, approval, annoyance, gratitude, disapproval, amusement, curiosity, love, optimism, disappointment, joy, realization, anger, sadness, confusion, caring, excitement, surprise, disgust, desire, fear, remorse, embarrassment, nervousness, pride, relief, grief*.

### 2.3 Annotation

Three raters were assigned to each comment, and asked to select those emotions, which they believed were expressed in the text. All three raters were native English speakers from India. The authors here rely on the results of a cross-cultural study showing that the emotion judgments of Indian and US English speakers largely occupy the same dimensions (Cowen et al., 2019). In the case where the annotators judged the text to be especially difficult to rate, they were able to choose not to assign any emotion to it. Whenever there was no agreement between the raters on a specific example, additional raters were assigned to it until each document was annotated at least twice with regards to the same emotional label.

### 2.4 Analysis

**Figure 1:**
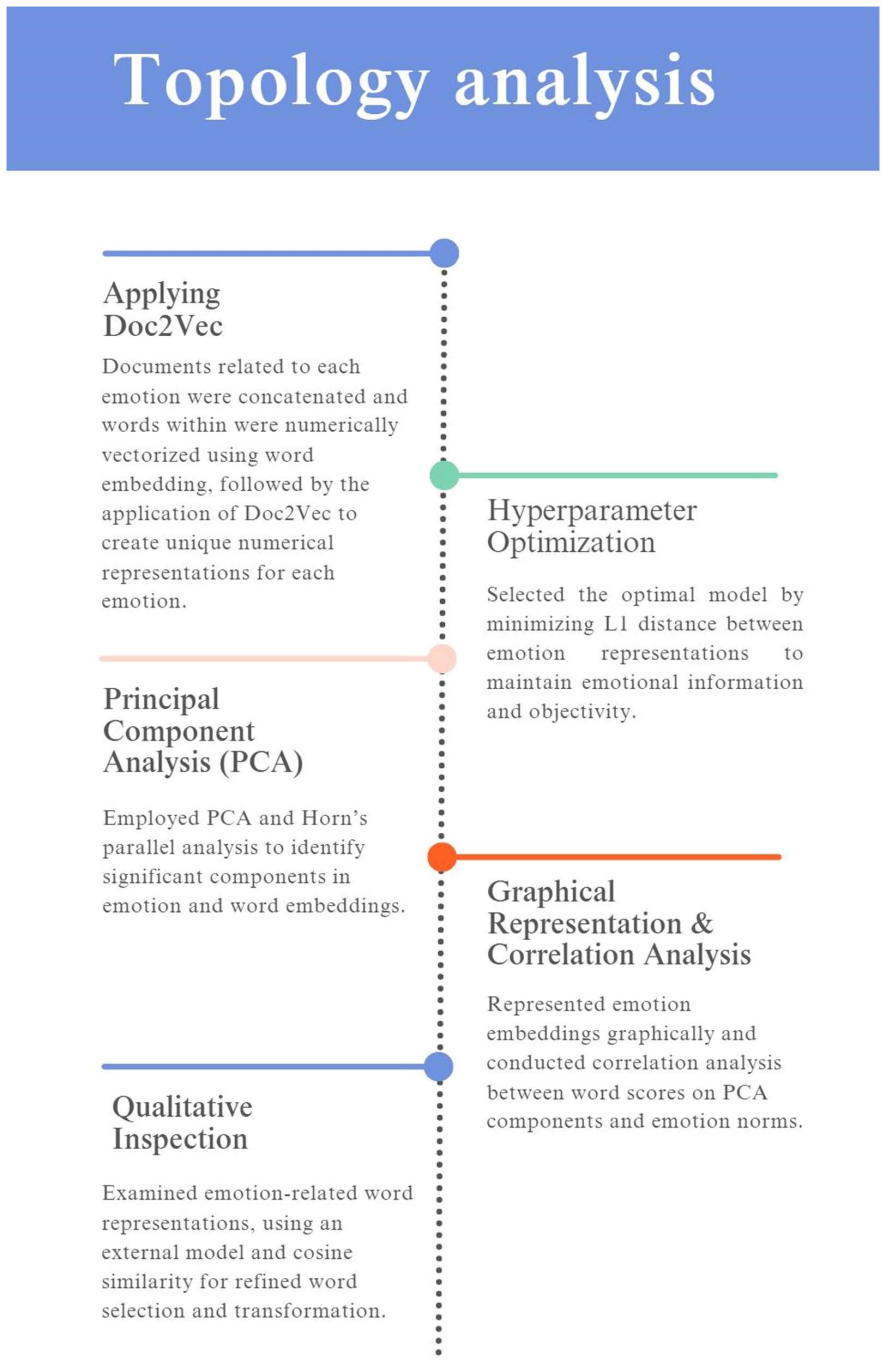
The steps of the analysis.

The analysis aims to represent the natural expression of emotions contained in the GoEmotions dataset in the word-embedding-based emotion space. The breakdown of the analysis is presented in Figure 1.

#### 2.4.1 Applying Doc2Vec to create numerical representations of emotions

The Doc2Vec algorithm (Le and Mikolov, 2014) was used to create emotion vectors for each emotion in the dataset. Documents that corresponded to a given emotion were concatenated into long documents, and then, during training, singular emotion vectors were created for each of these long documents. For a document to be judged as corresponding to a given emotion it was enough for it to be classified as so once. So, if a document was classified by two raters into two different emotions, this document then complemented two different concatenated series. This approach was chosen because applying majority voting retains less information from the annotators, and judging emotions is a highly subjective task where the objective truth can be rarely established. First, the words in each document were transformed into word vectors via a word embedding method, capturing the information embedded in each word. Then, these word vectors were used to build an emotion vector using the Doc2Vec algorithm, which treats the document as another word in the sentence and assigns numerical representations to it (Le and Mikolov, 2014). This resulted in a distinct numerical representation for each emotion that encapsulated the underlying sentiment, and thematic nuances present in the corresponding documents. Supplementary analyses of the distribution of document vectors and their relation to label centroids, including top-k nearest centroid accuracy, conducted to explore the resultant document vector space are presented in the Supplementary Material for the interested reader.

#### 2.4.2 Hyperparameter optimization

Because the Doc2Vec algorithm has a range of hyperparameters that had to be tuned in order to achieve the best representations, separate emotion spaces were created using different hyperparameter values. The hyperparameters that were taken into consideration were the collocation window size (5, 10, 20 words), minimum word count (10, 40, 60 words), embedding size (100, 200, 300, 400, 500, 600, 700, 800, 900 units). Every combination of the above parameters was tested. We chose the model that minimized the L1 distance between the emotion vectors to increase the likelihood that the emotion vectors represented meanings of emotions—as they would be more similar to each other if they truly belonged to the semantic space that describes emotions—while at the same time ensuring it did not impose any further predefined notions onto the contents of the vectors.

#### 2.4.3 Principal component analysis (PCA)

The emotion vectors were then subjected to a Principal Component Analysis, in line with the previous literature on decomposing emotions (Fontaine et al., 2002, 2007), which finds the dimensions along which the emotional representations (emotion vectors) vary the most and situates the emotions along them. The PCA was applied to the emotion vectors. Horn’s parallel analysis was used to determine the number of components that can be retained. This method compares the eigenvalues obtained from the factor analysis to those from a randomly generated dataset. If the eigenvalues from the factor analysis exceed those from the randomly generated dataset, the factors are considered significant and are retained.

#### 2.4.4 Graphical representation and correlation analysis

Emotion vectors were then plotted on a graph, and the words corresponding to the word vectors were tested for correlation with a set of words annotated with regard to their emotional loads along the first components (stipulated to be related to the components reported in the previous literature, Gendron and Feldman Barrett, 2009). In order to inspect these components, the word vectors retrieved from the dataset were transformed to align with the components identified by the PCA.

#### 2.4.5 Qualitative inspection

Because only some words present in the vocabulary were related to emotions, a qualitative inspection of only the highest and lowest-ranking words on each of the components could obscure the nature of the recovered dimensions, as it is the emotion related words that have the highest face validity when it comes to examining emotional dimensions. To circumvent this problem an external word embedding model with 300-dimensional vectors (Dadas, 2019) was used to sample the vocabulary for words related to the concept of emotions. The cosine similarity of word vectors was used to recover only 500 words most similar to the word vector for the word “emotion” based on the cosine similarity between the vectors that represented them. The resulting words were then subjected to the PCA transformation again, so that they could be evaluated qualitatively.

#### 2.4.6 t-Distributed stochastic neighbor embedding (t-SNE) analysis

To complement the Principal Component Analysis (PCA) and further explore the structure of the emotion vectors, we used t-Distributed Stochastic Neighbor Embedding (t-SNE). t-SNE is a nonlinear technique that helps visualize high-dimensional data by preserving local relationships, making it useful for identifying clusters and patterns that PCA might miss. For our analysis, we first standardized the emotion vectors to ensure that all features contributed equally. We applied t-SNE with the following settings: 2 components, a perplexity of 5, and a learning rate of 10. The random state was set to 22 to ensure that the results could be replicated. The perplexity was set to 5, the lower bound of the suggested values, due to the low number of emotion vectors. Perplexity, which balances attention between local and global aspects of the data, typically needs to be higher for larger datasets to capture broader relationships; however, for smaller datasets like ours, a lower perplexity is recommended as it helps maintain meaningful local structures (Van der Maaten and Hinton, 2008). The learning rate was set to 10, as this value provided a stable convergence during the embedding process, ensuring that the visualization accurately represented the underlying data patterns.

#### 2.4.7 Logistic regression on documents

To confirm the alignment of the PCA components with the emotional dimension of Valence, we recoded the original GoEmotions dataset from 28 emotions into positive and negative labels. The emotions classified as positive were admiration, love, gratitude, amusement, realization, optimism, curiosity, excitement, caring, joy, approval, pride, desire, and relief. The emotions classified as negative were sadness, disapproval, disappointment, annoyance, confusion, disgust, remorse, anger, grief, embarrassment, surprise, fear, and nervousness. If a text was labeled with a different emotion it was dropped. Here again, all text labels were taken into consideration and so if two annotators annotated a given text as joy, these were treated as separate rows. This approach was chosen over majority voting to preserve as much information from the original annotations as possible, given the subjective nature of emotion labeling. The final dataset consisted of 155,663 text—label pairs. We then transformed the document vectors from the Doc2Vec model using the PCA model previously fit on the emotion vectors, resulting in a four-dimensional vector for each document. These vectors were subsequently used in a logistic regression with the positive/negative labels as the dependent variable.

## 3 Results

### 3.1 Horn’s parallel analysis

**Figure 2:**
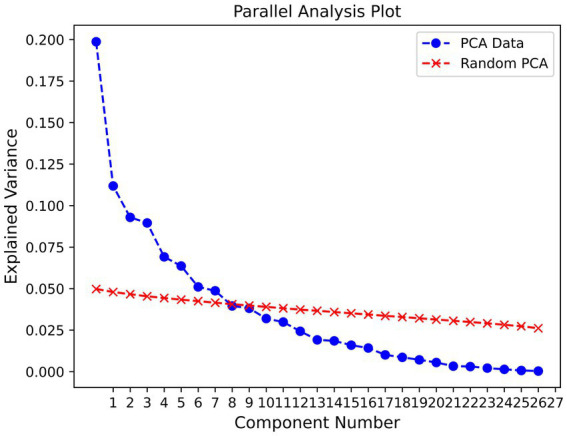
Parallel analysis plot.

The Horn’s parallel analysis indicated that the first seven components were significant and should be retained (see Figure 2). Even though seven components were significant, we chose to only inspect the first four of them, as after that number, the percentage of explained variance drops sharply.

### 3.2 Visualizing the emotion vectors

**Figure 3:**
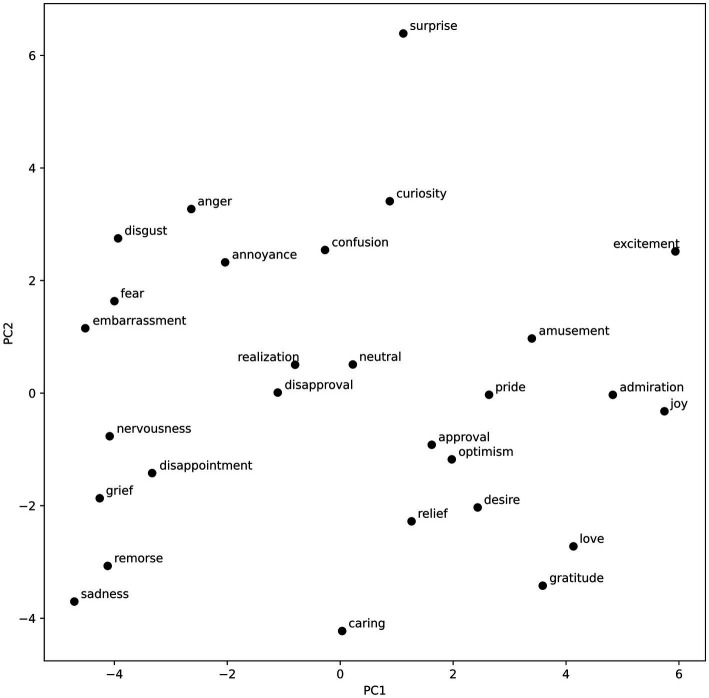
Emotional vectors plotted with regard to the first two PCA components.

**Figure 4:**
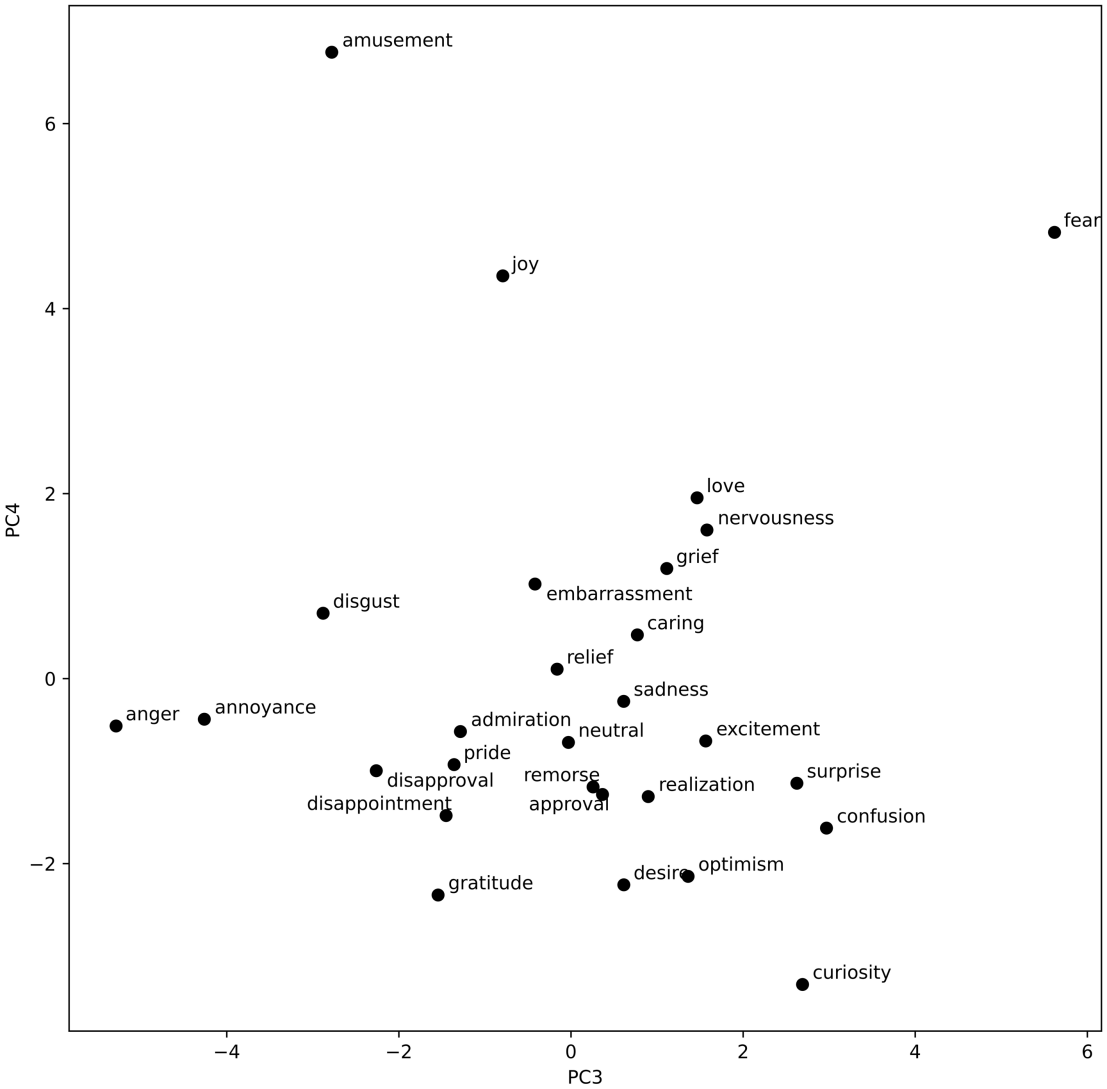
Emotional vectors plotted with regard to the third and fourth PCA components.

To visualize the emotion vectors regarding the components recovered by the PCA, we plotted them on two 2-dimensional graphs. The visualizations can be found in Figures 3, 4.

### 3.3 Correlation results

Due to the issues with word norm availability, only the first three components were checked for correlations with the emotional norms. The vocabulary of words from the GoEmotions dataset was filtered to remove the words that occur fewer than 50 times and more than 1,000 times in the dataset. From among those, 364 words overlapped with the norm dataset (Bradley and Lang, 1999), which consists of 1,030 words. The scores from the first PCA component achieved a correlation of *r =* 0.31 for valence with *p =* 2.48 × 10^−9^. The correlation of the second component and the norms for arousal were found to be insignificant with *r = −0.13, p =* 0.14. The third component was also insignificant for its correlation with dominance at *r = −0.02, p =* 0.68. As the quality of word vectors is heavily dependent on the amount of text on which they were trained, this analysis was not replicated in the robustness analysis.

### 3.4 Qualitative words inspection

The external word embedding model (Dadas, 2019) was then used to pick 500 words from the vocabulary, which had the highest cosine similarity with the word “emotion”. The numerical representations of words were then subjected to a PCA transformation. Finally, 30 highest and lowest words on each component were extracted (see Table 1). Again, as this analysis is word vector dependent, it was not replicated in the robustness analysis. For this check, we concentrated on the visual inspection of the emotion vectors. The overall positions of the emotion vectors on the PCA dimensions changed only slightly, which we attribute to the lower number of datapoints in the split datasets.

**Table 1:** Highest and lowest ranked words for each of the PCA dimensions.

PCA dimension	Words
PCA 1 high	together, fun, play, character, music, hate, story, interesting, amazing, album, especially, surprise, would, song, characters, interest, wish, unfortunately, perspective, love, stuff, one, happens, much, learned, ideas, quite, filled, sound, change
PCA 1 low	behavior, somehow, without, nobody, body, pain, almost, meant, happened, cause, clearly, completely, funny, away, humans, wrong, nothing, hurt, brain, others, trust, feel, saying, thinking, situation, someone, caused, truth, honestly, must
PCA 2 high	interesting, religion, seen, actually, basically, picture, crazy, different, even, clearly, wonder, literally, political, talking, beyond, individual, rather, actual, behavior, look, almost, quite, people, irony, pure, another, would, incredibly, power, exactly
PCA 2 low	pain, feel, feeling, appreciate, hear, alone, sometimes, hope, situation, life, better, good, feelings, felt, heart, true, always, everything, laugh, bad, able, thoughts, wonderful, choice, whatever, relationship, focus, anyway, loved, wish
PCA 3 high	story, happen, might, different, interesting, hope, scared, could, someone, something, weird, anyone, hear, totally, happened, never, afraid, crazy, bring, imagine, quite, would, moment, bit, alone, similar, true, surprise, nobody, together
PCA 3 low	give, good, say, literally, trying, opinion, people, words, saying, word, idea, understand, absolutely, mean, bad, play, incredibly, sound, everything, strong, either, power, behavior, move, point, every, reasons, everyone, telling, nothing
PCA 4 high	tell, imagine, moment, sad, sometimes, everyone, kinda, crying, loud, remember, little, fun, somehow, even, angry, someone, funny, feel, triggered, almost, thought, cry, still, tears, honestly, scene, seeing, hurt, scared, turn
PCA 4 low	interesting, change, give, need, opinion, anything, faith, unfortunately, means, different, situation, anyone, given, heard, deal, question, quite, rather, great, yet, something, individual, often, knowledge, hear, move, talent, nothing, however, another

### 3.5 Robustness check

To analyze the robustness of our analysis we additionally randomly split the dataset into two equal halves and repeated the analysis described in the Method section on these two halves, to ensure that similar distributions of emotion vectors are achieved. The overall positions of the emotion vectors on the PCA dimensions changed only slightly, which we attribute to the lower number of datapoints in the split datasets. The full report of the robustness check can be found in Supplementary materials.

### 3.6 t-SNE components visualization

**Figure 5:**
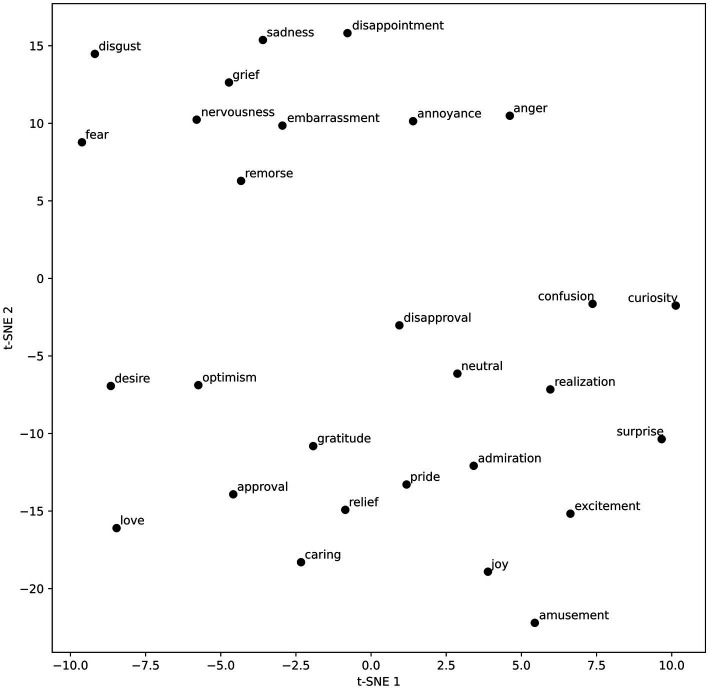
t-SNE components.

The results of the t-SNE analysis were plotted in Figure 5.

### 3.7 The logistic regression

The only significant variable in the regression model was the first PCA component (*β* = 1.60; *p <* 0.001; see Table 2).

**Table 2:** Logistic regression results predicting sentiment for texts.

Variable	B	SE	z	*p*	95% CI
Constant	−0.401	0.259	−1.551	0.121	[−0.909, 0.106]
PCA 1	1.599	0.209	7.657	0.000	[1.189, 2.008]
PCA 2	−0.116	0.251	−0.462	0.644	[−0.609, 0.377]
PCA 3	−0.466	0.359	−1.297	0.195	[−1.170, 0.238]
PCA 4	−0.254	0.318	−0.801	0.423	[−0.877, 0.368]

Dependent variable: sentiment. Observations: 155,633. Pseudo R-squared: 0.0003071. Log-Likelihood: −103,800. LLR *p*-value: 4.684e−13.

## 4 Discussion

The visualization of the emotion vectors (see Figure 3) along the first component complies with the valence negative–positive dichotomy. On the right, there are many high valence emotions such as joy, admiration, excitement, gratitude, love, and amusement. On the left, negative low-valence emotions can be found. These include disgust, fear, embarrassment, nervousness, disappointment, grief, remorse, and sadness. The second component seems to reflect the arousal dimension, with high scores assigned to such emotions as surprise, curiosity, anger, excitement, disgust, and annoyance; and low scores assigned to caring, gratitude, sadness, remorse, grief, and relief. Interestingly, love and desire are also classified among low arousal emotions. This could be an artifact of the nature of the dataset, and the fact that posts classified as love and desire could in many instances relate to those emotions being not satisfied, and thus including words that usually would be associated with sadness, and other low valence, low arousal emotions. Another possibility is that, purely due to the nature of the PCA, the first component does not fully capture the valence spectrum; however, the arrangement of the rest of the emotions enables a partial identification with the valence dimension. The third component (see Figure 4) is a lot less varied, with a lot of emotions clustered in the middle. Considering that it explains less than 10% of the variance in the word vectors, that is to be expected. This component, however, clearly separates such emotions as anger, and annoyance (high dominance) from emotions such as fear, curiosity, and confusion (low dominance). The fourth component, explaining the least amount of variance, could reflect the fourth dimension of emotional experience, namely unpredictability. This is evidenced in the strict separation of curiosity from amusement. However, the emotion of fear does not match this interpretation, and thus, it is not possible to state it with certainty. The distribution of emotion vectors was largely replicated during the robustness analysis for the first two dimensions (valence and arousal). The last two dimensions were significantly less pronounced, which is most probably the effect of smaller datasets, as each of the two datasets contained only half of all the text available for the primary analysis (see Supplementary materials).

The results of the correlation tests of the placement of words alongside the different components have to be interpreted in the light of the fact that while emotion vectors synthesize the information from many documents, labeled with a given emotion, single word vectors only relate to a limited number of nearest words on each side of the specific token. This results in the word vectors carrying less information and thus not being a robust indicator of emotional expression. Still, even under these strict limitations, the first component achieves a robust correlation with its corresponding norm (*r =* 0.31; *p* < 0.001). It should be noted that the lack of significance of the other components does not prove that they are not related to their corresponding dimensions. This point is underlined by the scarcity of information embedded in their respective word vectors, as well as the fact that only a small portion of words from the GoEmotions dataset actually overlap with the available norms (Bradley and Lang, 1999).

Even though the qualitative inspection of words suffers from the same limitation of word vectors carrying less information than the emotion vectors, some interesting examples that corroborate the correlation between principal components and the dimensions of emotional experience can be found (see Table 1). Scored high on the first component (reflecting valence), are such words as together, fun, play, music, interesting, and love, all of which relate to high valence concepts. On the other side of the same component, there are words like pain, and hurt, both related to low valence concepts. For the second component, high scoring are words like interesting, crazy, power, and incredibly, which relate to high arousal; low scoring words are feeling, and alone, reflecting low arousal. The high scoring words on the third component are among others: scared, afraid, crazy, and surprise corresponding to low dominance; lower-scoring words on this component are words like absolutely, strong, power, and incredibly, reflecting high dominance (keep in mind that in the case of the third component the factor loadings are stipulated to be negatively related to the dominance dimension; see Figure 4). Finally, the words presented for the fourth component do not seem directly related to the dimension of unpredictability.

The aforementioned words mostly confirm the relation of the PCA components to the emotional dimensions; however, as can be seen from Table 1, not all of the presented words fit this pattern. Examples such as hate for high valence, laugh for low valence, wonderful, and laugh for low arousal do not fit into the outlined interpretation. These outliers could exist both due to the aforementioned problem with low informative value of specific word vectors and due to the specific ways in which they were used in their corresponding posts. Because of the high volume of the dataset, a qualitative exploration of each and every post within which they were found is impossible.

The t-SNE analysis revealed two main clusters of emotion vectors (see Figure 5). One cluster comprises negative emotions such as anger, sadness, disappointment, and remorse. The other cluster includes mainly positive emotions such as admiration, pride, excitement, joy, and amusement, as well as neutral emotions. Interestingly, disapproval, an openly non-positive emotion, is also found in this cluster. This bipolar structure confirms a significant influence of the valence dimension on the semantic arrangement of the emotion vectors. Since t-SNE focuses on preserving pairwise distances between data points (Van der Maaten and Hinton, 2008), it primarily reflects the valence dimension, while the other dimensions identified by PCA are not visible in the t-SNE visualization, as expected. Consistent with t-SNE’s objective, emotions with similar meanings and expressions (e.g., desire and optimism; confusion and curiosity; sadness and disappointment) are positioned close to each other. It is important to note that the t-SNE results are sensitive to the choice of hyperparameters. In this analysis, we selected parameters that clearly delineated clusters, but different settings could produce varying results. A comprehensive exploration of all possible hyperparameters is beyond the scope of this paper.

Finally, the logistic regression results indicated that the first PCA component has a significant relationship with the sentiment of the texts (*β* = 1.60, *p* < 0.001; see Table 2). This finding further corroborates the conclusion that the first component reflects the valence dimension. Although the amount of variance explained by the model is very low (Pseudo R-squared: 0.0003071), this is expected because the emotion vectors used to create the PCA components were derived from the compressed information of over 50,000 texts, making it impossible to retain all information about every single text. Similar to the situation with the word vectors, the individual texts were only small snippets of the long-concatenated series that generated the emotion vectors.

### 4.1 Limitations

Our methodology assumes that words surrounding a specific token are indicative of its emotional connotation. However, this assumption does not consider the complexity of language and semantics. The emotional connotation of a word can significantly change depending on its position and usage in the sentence. As a result, single-word vectors may carry less information and be less reliable indicators of emotional expression. This challenge is reflected in our correlation test results, which, while statistically significant, show a relatively low correlation coefficient (*r* = 0.31). While more advanced word embedding methods that consider distant relations between words exist, such as transformer models (Vaswani et al., 2017), they are limited in the length of the text that they can represent, and thus are not sufficient for the current task where long, concatenated texts were analyzed. One possibility of using them is to average the vectors representing texts related to specific emotions, however, due to the noise inherent in this averaging, this method was not chosen for the current study.

Additionally, the interpretations of the third and fourth components of the PCA analysis might not fully correspond to the emotional dimensions of dominance and unpredictability, respectively. The third component was less varied and mainly clustered around the middle, suggesting a limited variability in dominance among the emotions. The fourth component explained the least amount of variance and its link to the dimension of unpredictability was inconclusive, especially given the unexpected positioning of certain emotions such as fear. Furthermore, there were certain word examples that did not fit the expected emotional dimensions, such as ‘hate’ for high valence and ‘wonderful’ for low arousal. While we attribute these anomalies to discourse-related artifacts and noise, they may also point to the complexity and multidimensionality of emotions that a linear component analysis may not fully capture. Another possibility points back to the information issues related to analyzing single word vectors, as they carry significantly less information than their emotion vectors counterparts.

From the methodological perspective, the fact that the emotions were labeled by the readers of text, and not their authors, stands in disagreement with the methods of previous studies, which often probed the person who experienced the emotions directly for their descriptions. One cannot expect that in all possible cases the annotator will correctly judge the emotion of the writer, or that the writer will always honestly describe their internal affairs. While the question of whether the influence of these two confounders is strong enough to produce qualitatively different results is an open one, the problem of text-based emotion communication and understanding is important in itself. This is especially true in the current age, where a lot of communication is done through text.

The preset number of emotion labels can also be seen as a limitation in the sense that by using them, the results of the current study will be biased by previous literature that has produced them. On the other hand, if annotators had been asked to describe the emotions in an open-ended manner, their results would still have to be categorized into label-like groups just the same. This grouping would be necessary to bind enough different texts together to produce robust emotion vectors. Drawing from the knowledge generated by previous studies is therefore a defensible alternative.

Finally, it is worth mentioning, that while the research on emotional components has a long history (Gendron and Feldman Barrett, 2009), the current study is to our best knowledge the first attempt at recreating emotional components based on numerical representations of natural language and, as such, is to be viewed as exploratory research. The findings of this study are best viewed as an invitation to use word embeddings to study psychological phenomena using newer, better-suited methods that allow researchers to analyze qualitative data in a quantitative manner.

### 4.2 Implications

Despite its exploratory nature, the current study shows that similar emotional components to the ones presented by the previous literature can be extracted from text using word embeddings. Specifically, these components were recovered by triangulating the semantic content of texts sourced from social media with peoples’ judgements of what emotion the author of these texts wanted to express (limited to the 28 emotions picked for annotation). Considering the two confounders present—first the willingness of the author to honestly communicate their emotions, and second, the ability of the annotator to correctly gauge what the author wanted to communicate—it is difficult to claim that the topology reported in the current study perfectly reflects the topology of internal emotional experience. Furthermore, given that the annotators were limited in their responses to a preset list of 28 emotions based on psychological literature, this study cannot introduce novel emotional phenomena, as it is constrained to those studied by previous researchers.

However, what this study shows is that the defining dimensions of emotions, as studied through more direct, yet less ecologically valid means of questionnaires and self-reports, are reflected in the semantic structure of how they can be expressed in written language. This can be explained by the process through which our need to communicate our internal states through language shapes and creates language itself. This interpretation aligns with Chafe’s work, which emphasizes that the structure and use of language are deeply influenced by the need to communicate conscious experiences and suggests that our expressions in written language naturally reflect the dimensions of internal emotional states (Chafe, 1996, 2013).

This method, when compared to the previous studies which mostly used specialized questionnaires, allows for a more ecologically valid analysis of the core dimensions of emotions. It ensures that the extracted components are grounded in the naturalistic expression of emotions and not artificially constrained by the assumptions of any particular theoretical model (Jackson et al., 2022). However, due to the indirect procurement of emotion labels (through readers and not directly from the authors), as well as the noise present in naturalistic expressions, it does not directly challenge existing methods, complementing them instead.

However, the presence of this noise could shed some light on the differences between the previous studies in the number of components that can be recovered (Bliss-Moreau et al., 2020; Fontaine et al., 2007; Mehrabian, 1996). This is evidenced by the clear dichotomy between fear and anger on the third component, supported in part by the qualitative word inspection, and by the vague sketch of unpredictability on the fourth of the recovered components. Perhaps with cleaner data and higher sample sizes, these components could be systematically recovered using classical methods. Another possibility is that laboratory studies obscure certain dimensions of emotional experience. This could be true especially for the dimension of dominance, the expression of which could be socially undesirable. Here the use of external annotators, rather than the authors of the text becomes an asset as it eradicates the influence of such social undesirability on the effects of the study.

As a last point, it is important to emphasize that the “emotion vectors” discussed in this study are purely mathematical representations derived from word embeddings, capturing the semantic and emotional content of text (Gutiérrez and Keith, 2019; Mikolov et al., 2013a,b). Unlike vectors of force in physics, which have a direction and magnitude related to physical movement, emotion vectors do not directly correspond to any physical or embodied experiences. They are abstract, numerical constructs designed to encapsulate the relationships between words in a multidimensional space, reflecting the latent structure of emotional content in language. This distinction is crucial to avoid conflating these computational representations with the physiological or psychological processes involved in action readiness, which pertains to the body’s preparation for specific actions in response to emotions (Frijda, 2010). Nonetheless, this separation does not diminish the potential value of exploring how these numerical representations might correlate with or illuminate aspects of embodied emotions. Future research could delve deeper into this intersection, investigating how emotion vectors could be used to study the embodiment of emotions, perhaps by correlating these computational measures with physiological data or by incorporating word embedding techniques into previous studies that tested the influence of text data on participants’ action-readiness (Lewinski et al., 2016). Such explorations could provide a richer, more integrated understanding of how emotions are represented and experienced.

### 4.3 Future directions

Future studies could try to recreate the current study on additional datasets of comparable quality. This would require researchers to assemble datasets of adequate length and content variance. The task of systematizing such endeavors has not been undertaken yet; however the great work done by Google (Demszky et al., 2020) can offer some directions in that regard. To our knowledge, as of yet, no dataset of comparable quality exists in open access. However, the data itself is available on the Internet, and its size is constantly growing, due to the popularity of social media sites.

Alternatively, recreating this study on a dataset with emotions annotated by the text authors instead of readers, could provide valuable information on the nature of the difference between these two emotional planes. This kind of research could shed more light on the problems related to communicating emotional information over the internet and through other text-based media, with an emphasis on the different sources of noise that partake in this process and can in many cases result in misunderstandings. The method itself can also be extended to different domains of psychology. For example, it could be well applied to the task of reconstructing the components of personality, assuming that the data are found to support this endeavor. Word embeddings can also be used in a completely data-driven way to analyze the results of qualitative interviews and create completely new psychological constructs. Furthermore, the method bypasses the difficulties in analyzing the emotional experience of individuals associated with such limitations as memory bias in answering questionnaires. The possibility of analyzing the text written by a specific individual over a span of time could therefore allow researchers to get a glimpse of what so far has been hidden behind population-wide studies—the way people express and experience emotions individually.

From a technical perspective, there is a possibility that the method of creating emotion vectors and applying PCA to them with the aim to extract emotional dimension components could be repurposed as a feature extraction method for emotion prediction. Future studies could try to apply similar techniques to this and other datasets and see whether the addition of these extracted features to more advanced machine learning models, such as deep learning architectures, XGBoost, SVM with non-linear kernels, and artificial neural networks (ANNs) leads to improved model accuracy and robustness.

## Data Availability

Publicly available datasets were analyzed in this study. This data can be found at: https://github.com/hplisiecki/emotion_topology.
