# Supplementary material for: Emotion topology: extracting fundamental components of emotions from text using word embeddings
Source: Front Psychol. 2024 Oct 8;15:1401084. doi: 10.3389/fpsyg.2024.1401084 (PMC11494860; doi:10.3389/fpsyg.2024.1401084)
Supplement: Supplementary file 1 [file Data_Sheet_1.docx]

Supplementary Material

# Emotion Topology Robustness Tests

To estimate the stability of the recovered components for different subsets of the Goemotions dataset we have split randomly into two equal parts and recreated the PCA based analysis reported in the main manuscript. Due to the lower amount of data in each of the two test datasets we do not expect the components found in the main manuscript to be reconstructed completely. Rather, we want to estimate the degree of their stability.

As can be seen from the Figures 1 and 3, the valence component seems to be stable across the two splits, with positive emotions such as joy, love and gratitude situated on one side of the first PCA dimension, and negative emotions such as anger, disgust, and annoyance on the other side. As for the second PCA dimension, supposedly corresponding to arousal – here again the component seems stable with high arousal words such as amusement, and surprise on one side of the dimension, and calmer emotions on the other such as caring, remorse, and optimism. As for the third dimension (see Figures 2, and 4), expected to represent the dominance dimension, it is definitely less delineated than in the main analysis. It seems to be recreated to some extent in the second corpus, where anger dominates the left side of the dimension, and fear rests just to the right of the center. However, in the case of the first corpus anger and fear are much closer to each other which puts the reconstruction of dominance into doubt. As pointed out earlier in the main manuscript, the elusive nature of the third component mirrors its inconsistent findings in previous research. Finally, the fourth component is somewhat reconstructed, with curiosity, confusion, and surprise on the one extreme, and emotions such as joy, anger, amusement, and gratitude on the other. This is quite surprising as the dimension of unpredictability proved quite noisy in the original analysis.

Overall, while all of the dimensions have not been fully reconstructed from the subsets of the main dataset, the reconstruction of the two which were the most stable in past literature (valence and arousal), as well as partial reconstruction of the third and fourth component bolsters the claim of the main manuscript. Namely, that the fundamental components of emotions can be reconstructed from text, using word embeddings.

**Supplementary Figure 1.** First Corpus Components one and two

**Supplementary Figure 2.** First Corpus Components three and four

**Supplementary Figure 3.** Second Corpus Components one and two

**Supplementary Figure 4.** Second Corpus Components three and four

**2. Document embeddings label centroid correspondence analysis**

First, the documents from the goemotion dataset were categorized into specific emotion categories based on majority voting, rearing 47379 classified documents. The number of classified documents was smaller than the total number of unique documents in the dataset (57842) as 10463 documents did not have a majority emotional class. This is congruent with the distribution of labels reported by the Google team as these left-over documents usually had a pair of at least two labels which had more than one label annotated twice. For example, one such left over document was annotated twice for gratitude, and twice for hope, making it impossible to extract a majority label.

The majority annotated documents were then used to compute label-based centroids for each of the 28 emotions by averaging respective documents. To analyze the distribution of the document vectors in the vector space in terms of how they relate to the placement of the centroids we have run two analyses. The first one estimated the performance of a classificator based on the L1 distance between the label centroids and unique document vectors. The second one assumed that even though the nearest centroid label might not have been the correct label per a given document, the correct centroid might have been nonetheless still close to the vector, leading us to conduct a top-k nearest analysis where we gradually relax the assumption that the nearest centroid is the correct one, instead relying on 2 nearest, 3 nearest, and so on until 28 nearest. Both of these analyses were compared to the estimated performance of the classifier under the assumption that the placement of the centroids was completely random.

The performance of the first classifier was analyzed in terms of precision, recall, and the F1 score, rearing low performance of respectively 12.06, 17.34, and 11.12 (see Table 1.). While low, this performance is better both in the cases of precision and recall than the by chance estimated precision and recall which in both cases was 3.57. In both cases Macro averaging was used to aggregate the class-based precision and recall, giving equal weights to each class. The by chance F1 scored was not used because it does not provide meaningful insights in cases where the precision and recall are both low and similar, as it would simply reflect the poor performance seen in both metrics without adding any additional interpretative value. Instead, the focus was placed on comparing the actual classifier's performance to the baseline to demonstrate that, despite the low scores, the classifier was still able to capture some meaningful structure in the data beyond random chance. The precision, recall and F1 score, as well as the by chance metrics of precision and recall for individual classes is presented in Table 2. leading to the same conclusion.

| **Table 1.** | | |
| --- | --- | --- |
| ***Aggregated performance metrics for centroid analysis*** | | |
| **Metric** | **Macro** | **By Chance** |
| Precision | 12.06 | 3.57 |
| Recall | 17.34 | 3.57 |
| F1 Score | 11.12 | - |

| **Table 2.** | | | | | |
| --- | --- | --- | --- | --- | --- |
| **Label Specific Performance Metrics** | | | | | |
| **Label** | **Precision** | **Recall** | **F1 Score** | **Precision by Chance** | **Recall by Chance** |
| neutral | 0.52 | 0.11 | 0.18 | 0.34 | 0.04 |
| admiration | 0.35 | 0.21 | 0.26 | 0.11 | 0.04 |
| approval | 0.19 | 0.16 | 0.17 | 0.06 | 0.04 |
| amusement | 0.32 | 0.25 | 0.28 | 0.05 | 0.04 |
| gratitude | 0.21 | 0.17 | 0.19 | 0.05 | 0.04 |
| annoyance | 0.10 | 0.05 | 0.07 | 0.04 | 0.04 |
| curiosity | 0.12 | 0.06 | 0.08 | 0.04 | 0.04 |
| anger | 0.15 | 0.22 | 0.18 | 0.04 | 0.04 |
| disapproval | 0.08 | 0.07 | 0.07 | 0.03 | 0.04 |
| love | 0.16 | 0.23 | 0.19 | 0.03 | 0.04 |
| confusion | 0.09 | 0.15 | 0.11 | 0.03 | 0.04 |
| caring | 0.06 | 0.17 | 0.09 | 0.02 | 0.04 |
| disappointment | 0.07 | 0.08 | 0.07 | 0.02 | 0.04 |
| optimism | 0.11 | 0.26 | 0.15 | 0.02 | 0.04 |
| joy | 0.15 | 0.15 | 0.15 | 0.02 | 0.04 |
| excitement | 0.04 | 0.11 | 0.06 | 0.01 | 0.04 |
| sadness | 0.10 | 0.06 | 0.07 | 0.01 | 0.04 |
| surprise | 0.08 | 0.25 | 0.12 | 0.01 | 0.04 |
| realization | 0.04 | 0.09 | 0.05 | 0.01 | 0.04 |
| desire | 0.08 | 0.26 | 0.13 | 0.01 | 0.04 |
| fear | 0.17 | 0.07 | 0.10 | 0.01 | 0.04 |
| disgust | 0.06 | 0.27 | 0.10 | 0.01 | 0.04 |
| remorse | 0.07 | 0.31 | 0.11 | 0.01 | 0.04 |
| embarrassment | 0.02 | 0.08 | 0.03 | 0.00 | 0.04 |
| nervousness | 0.01 | 0.20 | 0.03 | 0.00 | 0.04 |
| relief | 0.02 | 0.17 | 0.03 | 0.00 | 0.04 |
| grief | 0.01 | 0.40 | 0.03 | 0.00 | 0.04 |

**Supplementary Figure 5.** The top-k accuracy of the centroid-based classification

The results of the top-k accuracy analysis (See Supplementary Figure 5.) shows that while the correspondence between the label centroids and the vectors that hold the same label is low when considering only the nearest centroid, it raises gradually beyond the increase assumed by the by-chance classification as the restriction imposed on the number of nearest centroids considered is relaxed (up to the inclusion of the 9^th^ centroid where the accuracy curve becomes parallel to the by-chance curve, and where the predictive value od the addition of the next centroids decreases below the addition value in the by-chance curve). This means that the overall distribution of the label centroids, and their respective document vectors is not random, and that their distribution is to a certain low extent affected by the semantic content related to emotional value.
